# Infection Control in Optometric Practice: Evaluating the Impact of Guidelines on Knowledge, Attitude and Practice Behaviours

**DOI:** 10.1007/s44402-026-00055-x

**Published:** 2026-03-16

**Authors:** Kerryn Hart, Sheela Kumaran, Nicole Carnt, Fiona Stapleton, Alexandra Jaworski

**Affiliations:** 1 Education and Research, Optometry Australia, Carlton, Australia; 2 School of Medicine, Faculty of Health, Deakin University, Waurn Ponds, Australia; 3 School of Optometry and Vision Science, Faculty of Medicine and Health, UNSW, Sydney, New South Wales Australia

**Keywords:** Attitudes, Guideline adherence, Health knowledge, Infection control, Optometrists, Practice guidelines as topic

## Abstract

**Purpose:**

Infection control (IC) is central to safe optometric care, yet little is known about optometrists’ knowledge, attitudes and practice behaviours (KAP) in this domain. This study aimed to assess the IC-related KAP of Australian optometrists and to evaluate whether engagement with newly released IC guidelines could improve these measures.

**Methods:**

This study comprised two components: a cross-sectional survey of Australian optometrists’ IC-related KAP (part A) and a pre-post intervention study assessing the impact of IC guidelines (part B). The survey, distributed in early 2021, included demographic items, KAP measures and explored perceived barriers to best practice and preferred educational resources. Knowledge was assessed using multiple-choice and true/false questions, while attitudes and behaviours were analysed using Rasch-scaled measures. Participants consenting to follow-up were randomised into control or intervention groups. The intervention group received updated IC guidelines before repeating the survey; the control group repeated the survey prior to guideline exposure.

**Results:**

A total of 302 valid responses were analysed in part A. Respondents demonstrated moderate knowledge (mean 58%), supportive attitudes (median 79/100) and reported enacting IC behaviours in practice (median 64/100), although notable gaps were identified in areas such as tonometer disinfection and infectious disease exclusion periods. Barriers included insufficient time (47%), workplace support (29%) and equipment access (28%). Trusted sources of IC information were Optometry Australia, government advice and clinical guidelines. In part B (*n* = 73), the intervention group demonstrated significant improvements in knowledge (*p* < 0.01) and attitude (*p* = 0.02) compared with controls, although the attitude change was not clinically meaningful. No significant changes were observed in self-reported behaviours.

**Conclusions:**

Engagement with IC guidelines significantly improved knowledge among Australian optometrists but did not translate into behaviour change. Findings highlight the need for multifaceted, theory-informed implementation strategies that address environmental and motivational barriers, alongside provision of trusted resources, to embed evidence-based IC practices in optometry.

Key Points

- This study provides the first comprehensive assessment of infection control knowledge, attitudes and practices among Australian optometrists.
- Exposure to infection control guidelines was associated with improved knowledge, which did not translate into significant changes in behaviour.
- Improving infection control practice in optometry will require multifaceted strategies that address behavioural, contextual and systemic barriers, as guidelines alone are insufficient.

## Introduction

Optometrists play a key role in safeguarding their patients, themselves and colleagues from the potential risks associated with infections [1]. It is hard to imagine a time in recent history when infection control (IC) was more front-of-mind, with the COVID-19 pandemic emphasising the importance of IC to practitioners and patients globally. During certain periods, optometrists were limited to providing urgent eyecare, with stringent IC protocols enforced [2]. This climate of heightened awareness of IC protocols provided a valuable and unique opportunity to explore the knowledge, attitudes and practice behaviours (KAP) of optometrists in Australia.

There have been many publications on IC KAP of health professionals [3–7]; however, there is scarce literature regarding optometrists. A small study in the USA [8] found several areas for potential improvement in optometrists’ IC practices, including enforcement of hand hygiene policies, use of personal protective equipment (PPE) when examining potentially infectious patients, disposal of eye-drop vials when contaminated and disinfection of tonometer probes. Two other studies based in Jordan [9] and Nigeria [10] specifically investigated knowledge and risk in relation to COVID-19. Although Jordanian optometrists were found to have basic knowledge, understanding and up-to-date information about the virus and preventative measures [9], their primary source of information was social media and only a quarter reviewed guidance from their professional association. A recommendation from this study was to improve access to appropriate IC guidelines for optometrists. The study involving Nigerian eye care professionals [10], including optometrists, ophthalmologists, opticians and ophthalmic nurses, focused on the knowledge of COVID-19 exposure risk and related IC guidelines. In contrast with Jordanian optometrists, a higher proportion of Nigerian optometrists reported that their professional association provided guidance during COVID-19. There is a current paucity of literature addressing the KAP of optometrists with regard to IC and the impact of IC guidance within the Australian setting.

One central question guiding this study was whether engaging with IC guidelines could enhance optometrists’ KAP. Gigueré et al. [11] found that printed education materials, including guidelines and peer-reviewed journal publications, improved healthcare professional practice, whereas limited pre-COVID-19 studies suggested that computerised materials had little impact [11]. This evidence predates the rapid digital adoption seen during the pandemic [12].

The primary aims of this research were to assess the current IC-related KAP of optometrists in Australia and to investigate if IC guidelines circulated electronically improve their IC-related KAP. The secondary aims were to identify the barriers to implementing best-practice IC, the perceived impact of COVID-19 on IC practice and which education resources were utilised by optometrists to guide IC practice.

## Methods

This study received Deakin University ethics approval (reference number: HEAG-H 238_2020). The study comprised two elements: part (A) a cross-sectional analysis of 2021 IC-related KAP among Australian optometrists and part (B) a pre-post interventional study aiming to determine whether IC guidelines could change KAP.

### Survey design

An online survey was developed and hosted on Qualtrics (Qualtrics, Inc., qualtrics.com). The survey comprised four sections: demographics, IC-related KAP, education resources used to drive IC behaviours and the perceived impact of COVID-19 on IC. The impacts of COVID-19 on IC for this population were reported elsewhere (unpublished conference abstract) and are not the focus of this paper. The survey structure is provided in Table 1.

**Table 1:** Survey structure.

Section	Number and types of questions/statements	Scoring
Demographics	8	N/A
Knowledge	15 – 11 multiple choice (single answer), one multiple choice (multiple answers), three true/false	• 1 point per correct response. One question had two correct responses. Total score was out of 16 and expressed as a percentage. Unanswered knowledge questions were marked as incorrect and awarded zero.
Attitude	7	• 5-point Likert scale: “Strongly agree (4) to Strongly disagree (0)
Practice behaviours	24	• All questions were on a 4-point scale: always (3), most of the time (2), some of the time (1), never (0) • Five had additional unsure and/or not applicable options • The ‘never’ option for four questions pertaining to expiry dates was framed as ‘never’ or ‘I don’t check expiry dates’
Other questions	Five—including questions on hand hygiene policies and barriers to providing best-practice IC	N/A
Education	Three—participants were asked to rank and rate the relative importance of education resources to inform their current and support their future IC practice	N/A

*IC* infection control, *N/A* not applicable.

The survey was based on a KAP survey of hospital staff [13] and surveys of optometric IC in other countries [8, 14]. It included questions that related to each of the main themes relevant to optometry in the Australian Guidelines for the Prevention and Control of Infection in Healthcare [15], namely hand hygiene, sharps handling, management of the physical environment, disinfection methods, reprocessing of reusable medical devices, respiratory hygiene, aseptic technique, waste management, transmission-based precautions and PPE. The survey was piloted by five optometrists who were not part of the study team, and minor amendments were subsequently made to the survey questions to improve clarity.

### Survey Distribution (Part A)

A link to the survey, along with a plain language statement, was distributed to members of Optometry Australia (the professional association for optometrists in Australia) via email in January 2021 and through Optometry Australia’s social media channels, including Facebook, Twitter and LinkedIn. In Australia, all optometrists must be registered with the Australian Health Practitioner Regulation Agency (Ahpra), the national regulatory body. At the time of recruitment, Optometry Australia had approximately 4900 members, representing approximately 80% of the 6175 Ahpra-registered optometrists in late 2020.

During the cross-sectional survey (part A), participants were invited to provide their contact details if they agreed to participate in a follow-up study evaluating the effectiveness of IC guidelines (part B).

### Intervention Study (Part B)

Participants who agreed to be contacted for follow-up were randomly assigned to either a control or intervention group (part B). The control group was sent an email to repeat the KAP section of the initial survey in March 2021, prior to publication of the updated IC guidelines [16]. The intervention group was sent an email with the link to the updated IC guidelines after they were published in April 2021. They were asked to complete the KAP section of the survey after reading the IC guidelines. Each group was instructed on whether they needed to read the IC guidelines before (intervention group) or after (control group) completing the survey.

### Scoring of Outcome Measures

Knowledge was assessed across multiple domains and summarised using composite scores and percentages. The attitude and behaviour scales were scored using Rasch analysis. The ordinal scores were transformed into interval measures using the Rasch Andrich Rating Scale Model with Winsteps software version 4.8.2 (Linacre, winsteps.com/winsteps.htm, 2021). Detailed psychometric properties of the scales are provided in Appendix 1. The scales were optimised by repairing disordered response categories and removing misfitting items. The final scales demonstrated unidimensionality and modest person reliability (≥0.70), sufficient to differentiate reliably between low and high performers. The Rasch scores (in logits) were rescaled on a 0–100 scale to facilitate comparisons and interpretation.

### Statistical Analysis

Descriptive statistics, including mean, average, medians, interquartile range (IQR), frequencies, percentages and 95% confidence intervals, were determined in GraphPad Prism 10.0.03 (Domatics, graphpad.com), Excel for Microsoft 365 (microsoft.com) or IBM SPSS version 29 (ibm.com). Differences between survey participants and the broader population of Ahpra-registered optometrists were assessed using two-proportion *z*-tests, comparing characteristics such as gender and therapeutic endorsement status, with *p*-values calculated for a one-sided test to determine whether the study proportions were significantly greater than those observed for the Ahpra population. Means and 95% confidence intervals (CI) were determined for knowledge data. Behaviour and attitude data were described by medians and interquartile ranges from the 0–100 scale determined through Rasch analysis.

Attitude and behaviour data for the intervention study were explored for outliers with box plots [17], by examining individual responses for survey questions and performing the robust regression with outlier removal (ROUT) method with *Q* = 1% [18]. Spearman rank correlation analyses were performed to evaluate relationships between KAP. Kruskal–Wallis and Mann–Whitney tests were used to explore differences in scores between groups, including control and treatment groups, at baseline and post intervention. The Fisher's exact test was used to identify if the intervention was associated with an improvement in knowledge score by at least 25%, which was regarded as clinically significant as it represented a participant answering at least three more questions accurately following the intervention. A change in attitude of at least 14.3% was regarded clinically significant as this could represent a change from strongly disagree to strongly agree (or vice versa) in one statement or a category change in more than half (four or more) of the attitude statements. A *p*-value ≤ 0.05 was regarded as statistically significant.

### Ethical Approval

This study received Deakin University ethics approval (reference number: HEAG-H 238_2020). Ethics approval included dissemination of deidentified results through peer-reviewed journal publication.

## Results

### Survey Responses

Of the 390 responses to the survey in part A, 302 responses were included for analyses. Almost a quarter of responses (*n* = 88) were removed from the analysis as they provided demographic information only (*n* = 78), did not indicate consent to participate (*n* = 3) or the respondents did not confirm they were Ahpra-registered (*n* = 7). The survey had a 6% response rate from Optometry Australia members which represented 5% of Ahpra-registered optometrists, consistent with similar surveys [19, 20]. Respondents were demographically similar to Ahpra-registered optometrists (Table 2), with higher participation from females (*Z* = 4.52, *p* < 0.01) and therapeutically endorsed optometrists (*Z* = 3.79, *p* < 0.01) in the survey.

**Table 2:** Demographics of study participant (parts A and B) and Australian Health Practitioner Regulation Agency (Ahpra)-registered optometrists [21].

Category	IC survey participants (Part A)	IC survey participants (Part B)	Ahpra^b^
Gender			
Male	92 (31)	20 (27)	2695 (44)
Female	210 (70)	53 (73)	3480 (56)
Age (years)			
29 and under	75 (25)	15 (21)	1525 (25)
30–39	70 (23)	20 (27)	1540 (25)
40–49	64 (21)	14 (19)	1252 (20)
50–59	59 (20)	14 (19)	1063 (17)
60–69	31 (10)	10 (14)	696 (11)
70 and over	3 (1)	0 (0)	99 (2)
Therapeutic endorsement^a^			
Yes	226 (75)	57 (79)	3962 (67)
No	74 (25)	15 (21)	2000 (33)
Primary practice location^a^			
Australian Capital Territory	5 (2)	0 (0)	107 (2)
New South Wales	93 (31)	25 (35)	2014 (33)
Northern Territory	1 (0)	0 (0)	34 (1)
Queensland	47 (18)	10 (14)	1264 (21)
South Australia	31 (10)	7 (10)	379 (6)
Tasmania	6 (2)	2 (3)	113 (2)
Victoria	99 (33)	20 (28)	1636 (27)
Western Australia	18 (6)	8 (11)	469 (8)

(numbers are provided with % in brackets)

*IC infection control*.

^a^*One participant in part B did not provide therapeutic endorsement or primary practice location demographics*.

^b^*Ahpra data from December 2020 is reported as this aligned best with the timing of the survey (January 2021)*.

Of the 302 original survey participants, 223 (74%) provided their details for follow-up. These respondents were randomised to either a control group (*n* = 111) or intervention group (*n* = 112). The follow-up survey was completed by 73 of the 223 respondents (33%), resulting in 49 controls and 24 interventions. Intention-to-treat analysis was applied to all data [22]. Four participants were excluded following ROUT analysis, leaving 48 controls and 21 interventions for analyses of attitude [17]. No outliers were identified for responses pertaining to behaviour.

### Cross-Sectional Analyses

#### Knowledge

A summary of participant responses for the knowledge component of the survey (correct responses and the most commonly selected incorrect responses) is presented in Appendix 2. Responses were evaluated against the recommendations in the Australian Guidelines for the Prevention and Control of Infection in Healthcare [15]. The total knowledge score ranged from 4 to 15 with an average score of 9.3 (95% CI 5.2–13.5) out of 16, equivalent to 58% (95% CI 33–84%). Approximately 18% of participants (*n* = 54) scored less than 50%.

#### Attitude

**Fig. 1:**
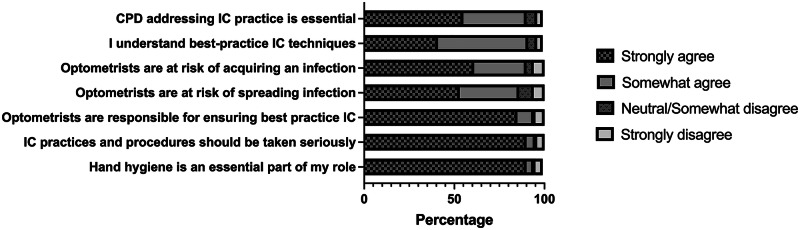
Optometrists’ attitudes towards infection control (IC).

Optometrists’ attitudes to IC are summarised in Fig. 1. The median score was 79 (IQR 68–88), indicating that most optometrists surveyed had a supportive attitude towards IC. Of note, at least 85% of optometrists strongly agreed that they are responsible for ensuring best practice IC, that IC practices should be taken seriously and that hand hygiene is an essential part of their role, but only 41% strongly agreed that they understood best practice IC techniques, and 55% strongly agreed that continuing professional development addressing IC practices is essential.

#### Practice Behaviours

Practice behaviours for the 18 items remaining after Rasch analysis are summarised in Appendix 3. The median score was 64 (IQR 58.25–71), indicating that most optometrists reported that IC was enacted in their clinical practice.

Less than half (48%, *n* = 146 of 292) of participants reported there was a written hand hygiene policy/guideline in place at their practice, and almost a quarter were unsure (23%, *n* = 69 of 292). When asked under what circumstances gloves were worn at the practice, the most common response was while examining patients with a suspected or known infection that is transmissible via tears (59%, *n* = 178 of 301). However, approximately a quarter of respondents incorrectly identified ‘3–5 days after initial infection’ as the recommended exclusion period if a healthcare worker has conjunctivitis [15]. A quarter of participants (*n* = 76 of 301) reported they would never use gloves and only 20% (*n* = 61 of 301) reported use of gloves when cleaning environmental surfaces. At least 87% reported they would dispose of a multidose topical drug bottle if it had passed its expiry date (*n* = 268 of 301) or if the tip came into contact with conjunctiva/lashes/skin/environmental surfaces (*n* = 262 of 301). Finally, the most commonly reported approaches to ensure a disinfected tonometer for every patient were to use disposable tips (54%, *n* = 162 of 302), wiping the probe’s surface with 70% isopropyl alcohol and air drying (48%, *n* = 146 of 302) and using a non-contact tonometer (41%, *n* = 124 of 302). The least common reported approaches were use of Tristel Duo OPH, a sodium chlorite disinfectant (Tristel Pty Ltd, tristel.com) (4%, *n* = 13 of 302) and soaking in dilute (1:10) sodium hypochlorite for 5–10 min (5%, *n* = 15 of 302). This aligned with the limited knowledge of most respondents in this area, who did not accurately identify the best-practice approach for tonometry probe disinfection using bleach [23] but instead opted for using hydrogen peroxide (39%, *n* = 119) or an alcohol wipe (31%, *n* = 92).

#### Correlations

Knowledge and attitude scores (*r* = 0.16, *p* < 0.01) and behaviour and attitude scores (*r* = 0.16, *p* < 0.01) were weakly positively correlated. There was no relationship between knowledge and self-reported practice behaviour (*r* = 0.03, *p *= 0.62). This was also the case for specific practice behaviours that directly investigated areas of knowledge. For example, knowing the correct duration for washing (*r* = 0.01, *p* = 0.84) or sanitising hands (*r* = 0.05, *p* = 0.42) was not correlated with always following the ‘five moments of hand hygiene’, a World Health Organization initiative identifying critical moments for hand hygiene during healthcare delivery [24]. Total knowledge score and an optometrist’s self-perceived understanding of best practice IC (question from attitude section) were not significantly correlated (*r* = 0.02, *p* = 0.78).

#### Barriers to Best-Practice IC

Twenty-five per cent of 301 respondents reported there were no barriers to providing best-practice IC. Of the remaining 226 respondents who reported at least one barrier, insufficient time (*n* = 140; 47%), insufficient workplace support (*n* = 88; 29%) and a need to purchase additional IC equipment (*n* = 84; 28%) were the most commonly reported barriers. Seventy-nine participants (26%) reported a lack of personal knowledge on IC as a barrier to providing optimal IC for patients.

#### Education

Two hundred and eighty-six participants completed this section. Advice from Optometry Australia (89%, *n* = 254), the government (86%, *n* = 246) and IC guidelines (83%, *n* = 236) were most commonly ranked ‘very important’ or ‘important’. In contrast, online forums (36%, *n* = 103), textbooks (36%, *n* = 103) and online search engines (30%, *n* = 85) were least commonly ranked ‘very important’ or ‘important’. Employer advice/directives were ranked ‘very important’ or ‘important’ by 75% (*n* = 214) of participants. Clinical guidelines were ranked as the most important IC resource, with websites that summarise IC research evidence, webinars/continuing education events and articles in Optometry Connection (an Optometry Australia print magazine) ranked second to fourth, respectively.

### Intervention Analysis

**Fig. 2:**
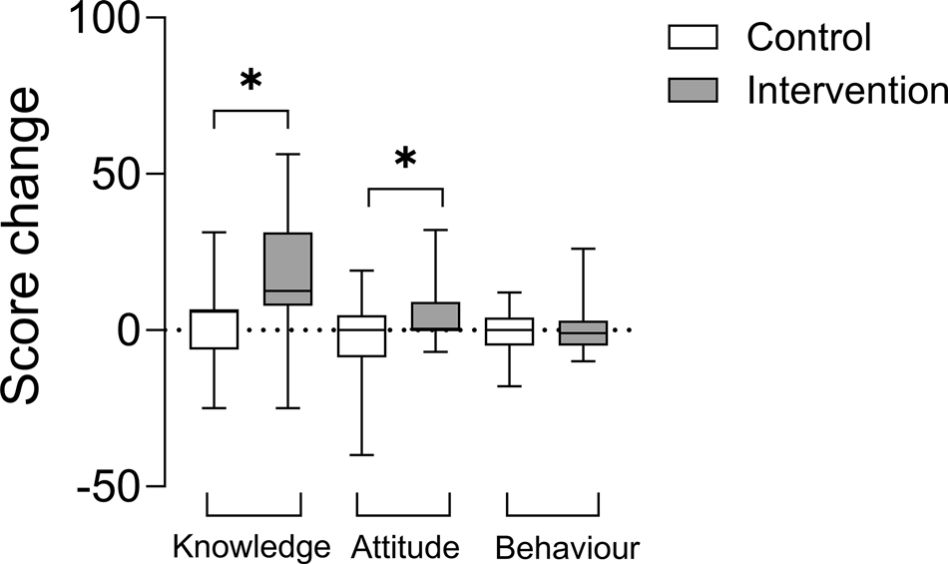
Boxplots showing changes (from baseline) in knowledge, attitude and behaviour scores (%) post intervention for the control and intervention groups. Knowledge and attitude scores improved significantly from baseline for the intervention group relative to the controls (*p* ≤ 0.02, asterisks).

Knowledge (*U* = 586.0, *p* = 0.98), attitude (*U* = 439.5, *p* = 0.39) and behaviour (*U* = 572.5, *p* = 0.86) scores were statistically equivalent between control and intervention groups at baseline (Table 3), despite differential response rates at follow-up. The change in knowledge (*U* = 891.5, *p* < 0.01) and attitude scores (*U* = 683.5, *p* = 0.02) improved significantly from baseline for the intervention group, relative to the controls, whilst behaviour appeared unchanged (*U* = 593.5, *p* = 0.95; Table 3 and Fig. 2).

**Table 3:** Median scores (%, interquartile ranges) for knowledge, attitude and practice behaviour of participating optometrists in the control and intervention groups at baseline and post-intervention.

	Baseline score (%)		Post-intervention score (%)	
	Control	Intervention	Control	Intervention
Knowledge	62.5 (50.0–68.8), *n* = 49	59.4 (50.0–69.5), *n* = 24	62.5 (56.3–68.8), *n* = 49	81.3 (65.6–93.8), *n* = 24
Attitude	79.0 (68.0–88.0), *n* = 48	76.0 (68.0–79.0), *n* = 21	73.0 (68.0–88.0), *n* = 48	79.0 (73.0–88.0), *n* = 21
Behaviour	63.0 (57.0–69.0), *n* = 49	61.5 (56.8–69.5), *n* = 24	61.0 (54.0–69.0), *n* = 49	62.0 (58.3–67.3), *n* = 24

Fisher’s exact test identified that more participants in the intervention group improved their knowledge score by at least 25% following the intervention, relative to the control group (*p* < 0.01). Fisher’s exact test showed that the proportion of participants who improved (*p* = 0.19) or negatively changed (*p* = 0.09) their attitude by 14.3% or more was the same in the control and intervention groups; thus, the difference in attitude between groups was not regarded as clinically significant.

## Discussion

This study uniquely captured the self-reported IC KAP of Australian optometrists, an area not explored previously. Importantly, it demonstrated that engaging with IC guidelines significantly improved knowledge, but although attitudes showed a statistically significant change, the difference was not clinically meaningful. No significant change was observed in self-reported practice behaviours. At baseline, participants demonstrated moderate knowledge of IC practices (mean score 58%), comparable to studies of other healthcare professionals [4, 7], with notable gaps in areas such as tonometer disinfection, exclusion periods and appropriate timing for hand washing. Although respondents expressed a sense of professional responsibility and generally supportive views toward IC, their perceived personal risk of transmitting or acquiring infection was relatively low. Similar patterns have been reported in other health professions [3, 25], where a strong sense of professional duty can still support suitable IC behaviours despite low perceived risk [25, 26]. These findings highlight a crucial evidence-practice gap and underscore the complexity of translating knowledge and attitudes into behavioural change in the optometric setting.

This gap is further illustrated by the self-reported behaviour data from the survey. Most optometrists reported good hygiene behaviours, with 85% performing hand hygiene between patients, above the benchmark figure of 80% set by the National Hand Hygiene Initiative in Australia [27]. However, adherence to some IC practices, such as taking infectious disease histories (24%) and isolating patients (18%), was low, whilst tonometry disinfection varied, with 61% of optometrists reporting use of high-level disinfection and 54% using disposable tips, but many still utilised alcohol wipes as the sole disinfection approach. As per findings for optometrists in the United States for tonometer probe disinfection, gaps remain in aligning reported practices with evidence-based best practices [23].

Improved knowledge following the intervention contrasted with attitudes, which showed a statistically significant but not clinically meaningful change. Unchanged behaviours, observed in this study, reflect a common challenge in implementation research where knowledge alone rarely translates directly into competence in clinical training [28]. Behavioural change theories [29–31], particularly the Capability Opportunity Motivation-Behaviour (COM-B) model, help explain these findings. While the guidelines may have enhanced psychological capability (knowledge), other determinants such as environmental opportunity (time, resources, practice policies) and motivational drivers were unlikely to be addressed through a single dissemination. This is supported by the weak correlation found between attitude and behaviour, consistent with the theory of planned behaviour, where beliefs, social/professional norms and perceived behavioural control can predict intention and action [32]. Addressing these broader influences may require multifaceted strategies [31, 33], including interventions that leverage experiential learning such as audit and feedback cycles.

Professional associations and guideline developers are well-positioned to support these strategies by providing not only credible information but also practical implementation support [34, 35]. In this study, respondents identified Optometry Australia, government resources and clinical guidelines as their most trusted sources of IC advice, consistent with findings in other eye care settings [10]. To improve adoption of best practices, professional bodies could embed tools such as checklists [36], promote peer-to-peer learning through communities of practice [37] and showcase clinical role models who demonstrate best practice; approaches known to enhance professional behaviour through social influence and experiential learning [38, 39]. These methods may address barriers such as time pressure, limited organisational support and resource constraints more effectively, which were identified in this study and previous research [7, 25, 35, 40], rather than relying on knowledge provision alone.

Therefore, future implementation of IC guidelines in optometry should consider a holistic, theory-informed approach that targets specific behaviours, not just knowledge or attitudes [41]. Behaviour change frameworks, such as the theoretical domains framework [30] and the behaviour change wheel underpinned by the COM-B model [31], can assist in designing strategies that overcome barriers and embed desired behaviours into daily clinical practice. For example, most participants in this study reported the absence of a hand hygiene policy or guidance at their practice, highlighting an environmental barrier that could inhibit implementation despite improved knowledge. Educational interventions should be multi-pronged. Fostering a culture of infection prevention and control (IPC), for example, by encouraging self-audits and reflections on IPC behaviours, may enhance adherence and accountability over time [42]. Indeed, a multifaceted approach is needed for organisational cultural change, with consideration of local contexts and vision for change and support from leadership [43]. For successful cultural change, support from both leaders and employees is key [43].

### Strengths and Limitations

The strength of this study lies in its robust survey methodology, including the use of Rasch analysis to enhance the measurement of attitudes and behaviours and its contribution to the limited literature on IC KAP among optometrists. The use of a controlled design with pre- and post-intervention surveys adds methodological rigour, and the national recruitment strategy enhances the generalisability of findings to the wider Australian optometry workforce.

Limitations include the reliance on self-reported data, which may overestimate positive practice behaviours of optometrists [44, 45] and introduce social desirability bias [46]. Furthermore, the attrition between surveys [47] and the short time frame between baseline and the intervention could explain the lack of change in attitudes and behaviours, as new habits can take four to 335 days to form [48], and changes in attitudes can lead to behaviour change [49]. Outliers in attitude responses suggested potential confusion with the survey scale. The knowledge section of the tool was not validated, and future studies could benefit from a bank of multiple-choice questions assessing different aspects of IC knowledge. Finally, the absence of longitudinal data limits understanding of optometrists’ knowledge retention over time.

The findings may be impacted by the imbalanced response rate between groups at follow-up. The intervention group was required to read IC guidelines in addition to completing the survey, which may have resulted in differential response rates due to this additional burden. However, baseline KAP scores were equivalent between groups (Table 3), supporting that differential response rates did not introduce systematic bias in key outcome variables.

A further limitation relates to the control group. While participants were invited to participate before the IC guidelines were formally released, there was a brief period during which the guidelines were publicly accessible online, and control participants could have accessed them if they chose to. Therefore, the study relied on participants adhering to instructions not to view the guidelines before survey completion. To account for this, an intention-to-treat analysis was used, preserving group allocation regardless of possible exposure and reducing the risk of bias [22].

Whilst findings are generalisable to the Australian optometry workforce, the study is specific to the Australian context, reflecting Australian IC guidelines and optometric practice standards. The applicability of findings to other jurisdictions may be limited, as IC guidelines, regulatory frameworks and professional practices vary internationally. Future research in other countries would be valuable to determine whether similar patterns of knowledge, attitudes and behaviours exist among optometrists in different healthcare systems and regulatory environments.

Further research is also needed to explore the reasons behind the under-utilisation of certain resources and to assess the effectiveness of different educational interventions in improving IC practices, including targeted training and implementing auditing and feedback mechanisms. Stakeholder engagement remains essential for identifying target behaviours and the barriers and enablers to their adoption [50].

## Conclusion

This study highlights opportunities to strengthen IC education and practitioner approaches in optometry. Understanding the interplay between knowledge, attitudes and practices is essential, requiring a holistic approach that couples knowledge acquisition with practical application and attitude change. Aligning resources and education with clinicians’ needs, improving access to trusted information and addressing implementation barriers can drive more effective IC practices, enhance patient safety and inform strategies for continuous improvement.

## Data Availability

The datasets generated and analysed during the current study are not publicly available due to restrictions imposed by the ethics approval and the need to protect participant confidentiality. De-identified data may be available from the corresponding author upon reasonable request and subject to institutional approval.

## Appendix 1: Psychometric Properties of the Scales Used for Rasch Analysis

Table 1 Rasch-based psychometric properties of infection control (IC) attitudes and behaviour scales.

**Table:**

Rasch property	Standards	Original attitude scale	Final attitude scale^a^	Original behaviour scale	Final behaviour scale^b^
Number of responses from cross-sectional and interventional study		375	375	375	375
Number of items		7	7	24	18
Number of response categories		5	4	4	3
Category thresholds	No disordering	Disordered (*N*, −0.64, −0.20, −0.99, 1.84)	Ordered (None, −1.54, −0.58, 2.11)	Disordered (*N*, −0.06, 0.06, 0.00)	Ordered (*N*, −1.28, 1.28)
Person Separation Index (PSI), Person Separation Reliability (PSR)	>2.0, >0.80	1.38, 0.65	1.54, 0.70	1.67, 0.74	1.87, 0.78
Wright’s Sample-independent Person (Test) Reliability based on maximum strata	≥0.8		0.93		0.97
Principal component analysis of residuals (PCA) Variance explained by Rasch measure	>50%	60.4%	56.7%	43.1%	45.5%
PCA, Eigen value of 1st contrast	<2	1.73	1.80	2.41	2.36
Item Infit - misfit	MNSQ (mean-square) <1.5	4 (1.41)	No misfit	4 items	No misfit
Item Outfit - misfit	MNSQ < 1.5	6 (1.56) 5 (1.47)	5 (1.48) 6 (1.42)	5 items	No misfit
Targeting	<1 logits	2.88	3.15	1.17	1.96
Measure range			−5.34 to 5.77	−4.74 to 4.74	−5.92 to 6.19

^a^Final attitude scale was derived by collapsing underutilised categories 1 (disagree) and 2 (neither agree/disagree). The PSI slightly improved with this model but remained sub-optimal. However, the Wright’s Sample-independent Person (Test) Reliability based on maximum strata was acceptable (>0.8). Deleting misfitting items 5 and 6 reduced PSI and was retained. There was no evidence of local dependence or multidimensionality.

^b^Final behaviour scale was derived by collapsing underutilised categories 1 (some of the time) and 2 (most of the time). Misfitting items 3–5,10,14 and 16 were iteratively deleted, improving PSI. These items mostly assessed access to facilities (e.g., hand wash, sinks etc) rather than behaviour. PCA of residuals indicated items 19–22 on expiry dates were clustering together. However, the disattenuated correlation between item clusters was 1, supporting the treatment of the scale as unidimensional for practical purposes.

## Appendix 2: Survey Responses Addressing Knowledge of Infection Control (IC) Principles

Table 2 Knowledge of infection control (IC) principles amongst Australian optometrists % (*n* ≤ 302). The percentage (and number) of optometrists indicating the correct response [15] and the most common incorrect responses are provided.

**Table:**

Question	% Correct response (*n*)	% Most common incorrect response (*n*)
Multiple choice questions		
What is considered the single most important strategy in preventing the spread of infection in optometry practice? (301)	Hand hygiene: 87 (263)	Slit lamp breath shields: 9 (28)
Covering the mouth/nose when coughing or sneezing should ideally be done with a: (301)	Tissue: 34 (103)	Flexed elbow: 64 (193)
If performing hand hygiene with hand wash (e.g., soap and water), the entire procedure should take: (301)	40–60 s: 22 (66)	20–30 s: 66 (200)
If performing hand hygiene with alcohol-based hand rub, the entire procedure should take: (300)	20–30 s: 61 (184)	5–10 s: 29 (88)
Which of the following statements about surgical masks & standard precautions is correct? (301)	Surgical masks should be used if a patient has a cold or influenza: 60 (179)	Surgical masks should only be replaced once every 4 h: 39 (116)
Other than Tristel Duo^a^, what else is considered a best-practice [23] tonometry probe disinfection option? (302)	Soaking in dilute (1:10) sodium hypochlorite (bleach) for 5–10 min, rinsing thoroughly and air drying: 30 (91)	Soaking in 3% hydrogen peroxide for 5–10 min, rinsing thoroughly and air drying: 39 (119)
What should be done with the cap of a multidose bottle while instilling drops in a patient’s eye? (302)	Hold cap in washed hand: 47 (143)	Place cap on tissue: 43 (130)
What is the recommended exclusion period if a healthcare worker has conjunctivitis? (301)	Until there is no discharge: 21 (63)	For 7–10 days after initial infection: 48 (143)
When a pathogen is transferred through a contaminated intermediate object, this type of transmission is referred to as: (301)	Fomite: 55 (166)	Direct contact: 29 (88)
It is recommended to disinfect RGP^b^ and soft contact lenses with: (302)	3% hydrogen peroxide: 66 (198)	Multipurpose solution: 28 (84)
The correct definition of disinfection is: (301)	A process that inactivates non-sporing infectious agents, using either thermal or chemical means: 36 (107)	A process that destroys all microorganisms on the surface of an instrument or device: 48 (145)
During standard precautions, which area(s) of the consulting room should be cleaned on a daily basis? (300)	Both correct responses: 84 (251) • ‘sinks/basins’ 85 (256) • ‘door knob/handle to consulting room’ 99 (296)	‘Walls’ or ‘walls and ceiling’: 6 (17)
True/False questions		
A foreign body needle can be disposed of in a normal waste basket, so long as the tip cover is replaced (i.e., it is re-sheathed). (302)	False: 89 (268)	True: 11 (34)
Disposable tonometer probes are considered biohazardous waste. (302)	True: 63 (190)	False: 37 (112)
Tonometer probes are considered semi-critical devices, so should undergo high-level disinfection. (302)	True: 82 (247)	False: 18 (55)

^a^Tristel Duo OPH = 100% sodium chlorite, citric acid, 1-Decanamine, N, N-dimethyl-N-oxide.

^b^RGP rigid gas permeable.

## Appendix 3: Survey Responses Addressing Infection Control (IC) Behaviour

Table 3 Self-reported practice behaviours of Australian optometrists % (*n* ≤ 301)^a^. The number of responses is provided in brackets.

**Table:**

Question	% Always (*n*)	% Most/some of the time (*n*)	% Never (*n*)
Is a history of infectious disease (e.g., HIV, hepatitis) sought from the patient (via patient history or questionnaire)? (299)	24 (72)	59 (175)	17 (52)
Does the practice evaluate employees’ adherence to hand hygiene? (301)	20 (59)	54 (163)	26 (79)
Is hand hygiene performed between each patient? (300)	85 (256)	14 (42)	1 (2)
Are the ‘five moments of hand hygiene’ [24] followed during each patient consultation? (299)	56 (166)	43 (130)	1 (3)
Is a face mask used if the patient has respiratory symptoms? (301)	75 (226)	24 (71)	1 (4)
Is a face mask used if you have respiratory symptoms? (300)	88 (263)	11 (33)	1 (4)
Is high-level disinfection used on instruments that come into contact with mucosal surfaces (e.g., tonometer probes, gonioscopes)? (300)	61 (183)	33 (99)	6 (18)
Patients with infective eye symptoms (i.e., discharge, conjunctivitis, etc.) are isolated from the rest of the practice (e.g., separate waiting room)? (301)	18 (53)	48 (144)	35 (104)
Patients with respiratory symptoms are isolated from the rest of the practice (e.g., separate waiting room)? (299)	25 (74)	44 (131)	31 (94)
Are topical drugs stored appropriately (e.g., manufacturer instructions stipulate Alcaine^b^ should be stored in the fridge; Mydriacyl^c^ should not)? (301)	59 (177)	36 (108)	5 (16)
Are single use drugs used once and then discarded? (287)^d^	48 (138)	48 (137)	4 (12)
Are saline vials used once and then discarded? (234)^d^	68 (159)	28 (66)	4 (9)
Are mydriatic drops used only when within expiry date? (295)^e^	82 (241)	18 (52)	1 (2)
Are topical anaesthetics used only when within expiry date? (296)^e^	85 (251)	15 (43)	1 (2)
Are staining agents (e.g., fluorescein/lissamine green) used only when within expiry date? (287)^e^	87 (250)	12 (35)	1 (2)
Is saline used only when within expiry date? (289)^e^	85 (245)	13 (39)	2 (5)
Are the appropriate environmental surfaces cleaned weekly (e.g., computer, keyboard and mouse; waste receptacle)? (293)^f^	70 (204)	29 (86)	1 (3)
Are the appropriate environmental surfaces cleaned daily (e.g., consulting room patient chair; hand washing sink)? (291)^f^	75 (218)	24 (70)	1 (3)

^a^Percentages were rounded to the nearest whole number, so totals are sometimes greater than 100%.

^b^Alcaine = proparacaine hydrochloride 0.5%.

^c^Mydriacyl = tropicamide 0.5% or 1%.

^d^Twenty-two per cent of participants (*n* = 66) reported the question about saline vials was not applicable and 5% (*n* = 14) reported the same for single use drugs.

^e^Some participants reported that they do not check expiry dates for mydriatic drops (1%, *n* = 3), topical anaesthetics (1%, *n* = 3), staining agent (4%, *n* = 11) and saline (3%, *n* = 9).

^f^Two per cent of participants (*n* = 6) were unsure if environmental surfaces were cleaned weekly or daily.
